# Omega-3 Fatty Acid Supplementation for Borderline Personality Disorder

**DOI:** 10.1192/j.eurpsy.2026.11001

**Published:** 2026-07-31

**Authors:** A. V. Samokhvalov

**Affiliations:** 1 Dr.Sam’s Health, Inc., Oakville; 2 Department of Psychiatry & Behavioral Neurosciences, McMaster University, Hamilton, Canada

## Abstract

**Introduction:**

Borderline personality disorder (BPD) is a highly prevalent and disabling condition associated with multiple psychiatric and somatic comorbidities and significant burden of disease. While many pharmacological agents have been tested for treatment of BPD [1], only few biological therapies are available, and the main treatment modality is Dialectical Behavior Therapy (DBT). At the same time, there have been clinical indications that certain nutrients may have a positive impact on the course of this condition [2]. Specifically, omega-3 fatty acids (O3FA) supplementation has shown some promise.

**Objectives:**

To evaluate the effects of omega-3 fatty acid supplementation in patients with BPD.

**Methods:**

Systematic review of literature.

**Results:**

A systematic literature search was conducted using a series of keywords capturing various O3FA- and BPD-related terms. The initial search yielded 755 entries. After removal of duplicates and title screening, 61 abstracts / full-text articles were reviewed. We have identified 5 studies directly investigating the effects of O3FA on various aspects of BPD during the search. Additionally, existing systematic reviews and meta-analyses were scanned for additional studies, and 2 more studies were added to the search. Full-text review of the included studies showed broad range of outcome measures. Most of the studies reported overall positive effects of O3FA on various clinical aspects of BPD such as impulsivity and affective dysregulation albeit with modest effect sizes.

**Conclusions:**

Limited data on clinical effects of O3FA supplementation for BPD demonstrated an overall positive albeit modest impact on multiple aspects of functioning in BPD. Our research suggests that O3FA supplementation can be safely used as an adjunctive treatment in patients with BPD and warrants further research in this area.

**Disclosure of Interest:**

None Declared

